# Frontiers of MicroRNA Signature in Non-small Cell Lung Cancer

**DOI:** 10.3389/fcell.2021.643942

**Published:** 2021-04-07

**Authors:** Xinping Zhu, Masahisa Kudo, Xiangjie Huang, Hehuan Sui, Haishan Tian, Carlo M. Croce, Ri Cui

**Affiliations:** ^1^Cancer and Anticancer Drug Research Center, School of Pharmaceutical Sciences, Wenzhou Medical University, Wenzhou, China; ^2^Comprehensive Cancer Center, Department of Cancer Biology and Genetics, The Ohio State University, Columbus, OH, United States

**Keywords:** NSCLC, miRNA, miRNA therapeutics, circulatory miRNAs, miRNA dysregulation

## Abstract

Lung cancer is the leading cause of cancer-related deaths worldwide and non-small cell lung cancer (NSCLC) accounts for more than 80% of all lung cancer cases. Recent advancements in diagnostic tools, surgical treatments, chemotherapies, and molecular targeted therapies that improved the therapeutic efficacy in NSCLC. However, the 5-years relative survival rate of NSCLC is only about 20% due to the inadequate screening methods and late onset of clinical symptoms. Dysregulation of microRNAs (miRNAs) was frequently observed in NSCLC and closely associated with NSCLC development, progression, and metastasis through regulating their target genes. In this review, we provide an updated overview of aberrant miRNA signature in NSCLC, and discuss the possibility of miRNAs becoming a diagnostic and therapeutic tool. We also discuss the possible causes of dysregulated miRNAs in NSCLC.

## Introduction

MiRNAs are a class of small single-stranded, endogenous non-coding RNAs with approximately 20~22 nucleotide length. miRNAs act as post-transcriptional gene regulators by binding to the complementary 3′- untranslated regions (3′-UTRs) of target mRNAs, resulting in translational inhibition or degradation of mRNAs (Bartel, 2009). The first miRNA, lin-4, was discovered in 1993 by Ambros and colleagues from the *Caenorhabditis elegans (C. elegans*) (Lee et al., 1993). In 2000, another miRNA in *C. elegans*, let-7, was reported by Reinhart et al. let-7 capable of being inhibited the expression of heterochronic gene lin-41 by sequencing specific RNA - RNA interaction with the 3′-UTRs of its mRNA (Pasquinelli et al., 2000; Reinhart et al., 2000). In 2002, Dr. Croce group provided the first evidence that miRNA was involved in human cancer pathogenesis. They revealed that miR-15a and miR-16-1, located at 13q14 chromosome region, were frequently deleted in B-cell chronic lymphocytic leukemia (CLL) (Calin et al., 2002). Over the past decade, growing evidences have indicated that the dysregulation of miRNAs is implicated in the development, progression, and metastasis of various cancers (Iorio and Croce, 2012a).

Lung cancer is the most commonly diagnosed cancer and the leading cause of cancer-related deaths worldwide (Bray et al., 2018). Lung cancer is classified into two main histological groups, including NSCLC (~85%) and small cell lung cancer (SCLC, ~15%) (Osmani et al., 2018). miRNAs dysregulation is frequently found in NSCLC, and aberrant expressions of miRNAs play a key role in NSCLC proliferation, invasion, and metastasis through regulating their target genes (Peng et al., 2013; Cortez et al., 2015; Liang et al., 2020). In this review, we briefly introduce aberrant miRNA signatures in NSCLC and summarize how miRNAs act as oncogene or tumor suppressor to regulate NSCLC progression and metastasis by modulating their target genes. We also discuss the possibility of miRNAs becoming therapeutic targets or biomarkers in NSCLC.

## Tumor Suppressor and Oncogenic miRNAs in NSCLC

miRNA dysregulation causes aberrant expression of their target genes, thus involves various aspects of cancer cells including cell growth, apoptosis, metabolism, and invasion (Bartel, 2004; Bracken et al., 2016). A number of studies have shown that miRNAs function as tumor suppressors or oncogenes in NSCLC by inhibiting their target genes (Table 1). Here, we introduce several prominent miRNAs that play critical roles in NSCLC.

**Table 1:** Representative oncogenic or tumor suppressive miRNAs in NSCLC.

**microRNA**	**Chromosome**	**Function**	**Expression**	**Targets**	**References**
Let-7a	9q22.3	Tumor-suppressor	Down-regulation	BCL2L1, IGF1R, CCND1, KRAS	Chin et al., 2008
	11q24.1				
	22q13.31				
Let-7b	22q13.31	Tumor-suppressor	Down-regulation	USP44, USP42, ATXN7L3, KRAS	Chin et al., 2008; Spolverini et al., 2017
Let-7c	21q21.1	Tumor-suppressor	Down-regulation	ITGB3, MAP4K3, TRIB2, USP44, USP42, ATXN7L3, TGFBR3	Kumar et al., 2014; Zhao et al., 2014; Spolverini et al., 2017
miR-486	8p11.21	Tumor-suppressor	Down-regulation	PI3KR1, IGF1R, IGF1, Pim-1, ARHGAP5, SNHG15, CDK14	Peng et al., 2013; Pang et al., 2014; Wang, J. et al., 2014; Jin et al., 2018
miR-134	14q32.31	Tumor-suppressor	Down-regulation	CCND1, EGFR	Qin et al., 2016
miR-218	4p15.31	Tumor-suppressor	Down-regulation	EGFR, Slug, ZEB2, IL-6R, JAK3, HMGB1	Zhang et al., 2013; Zhu et al., 2016; Shi et al., 2017; Yang et al., 2017
	5q34				
miR-326	11q13.4	Tumor-suppressor	Down-regulation	CCND1, Sp1	Sun et al., 2016; Wang et al., 2019
miR-34	1p36.22	Tumor-suppressor	Down-regulation	PD-L1, CDK4, HDM4, ZNF281, ZBP99, Snail1	Kim et al., 2011; Hahn et al., 2013; Okada et al., 2014; Cortez et al., 2015; Feng et al., 2019
	11q23.1				
miR-200	1p36.33	Tumor-suppressor	Down-regulation	ZEB1, ZEB2, PDL1, QKI, Foxf2, LOX, LOXL2, IL-8, CXCL1	Pecot et al., 2013; Chen et al., 2014; Kundu et al., 2016; Peng et al., 2017, 2019; Kim et al., 2019
	12p13.31				
miR-520	19q13.42	Tumor-suppressor	Down-regulation	VEGF	Zhou et al., 2019
miR-16	3q25.33	Tumor-suppressor	Down-regulation	MEK1, FEAT, Bcl-2	Chatterjee et al., 2015
miR-340	5q35.3	Tumor-suppressor	Down-regulation	PUM1, PUM2, ZNF503, CDK4, RAB27B	Fernandez et al., 2015
miR-26	3p22.2	Tumor-suppressor	Down-regulation	ITGβ8, HMGA1, MALT1	Chen et al., 2016
	2q35				
miR-196b	7p15.2	Oncogene	Up-regulation	GATA6, TSPAN12, FAS	Huang et al., 2020; Liang et al., 2020
		Tumor-suppressor	Down-regulation	Runx2, LIN28, FOS, UGT2A1	Tellez et al., 2016; Bai et al., 2017
miR-21	17q23.1	Oncogene	Up-regulation	PDCD4, SOCS1, SOCS6, PTEN, Apaf11, RhoB, Faslg, Spry1, Spry2, BTG2	Hatley et al., 2010; Zhang et al., 2010; Ma et al., 2011; Xue et al., 2016
miR-31	9p21.3	Oncogene	Up-regulation	CDK5, PTEN, p70S6K, ERK, AKT, RASA1, SPRY, SPRED	Meng et al., 2013; Edmonds et al., 2016
miR-224	Xq28	Oncogene	Up-regulation	TUSC3, CASP3, CASP7, TNFAIP1, SMAD4, p21, TXNIP, PTEN	Knoll et al., 2014; Wang et al., 2014; Cui et al., 2015a,b; Jeon et al., 2018
miR-155	21q21.3	Oncogene	Up-regulation	SOCS1, SOCS6, PTEN, TAB2, TP53	Xue et al., 2016; Van Roosbroeck et al., 2017; Wan et al., 2019
miR-1246	2q31.1	Oncogene	Up-regulation	MT1G, DR5	Kim et al., 2016; Yuan et al., 2016; Zhang et al., 2016
miR-210	11p15.5	Oncogene	Up-regulation	E2F3, SDHD, FGFRL1, VMP-1, RAD52,	Puisségur et al., 2011; Cui H. et al., 2015
miR-221/222	Xp11.3	Oncogene	Up-regulation	APAF1, SOCS3, TIMP2, TIMP3, PTEN, p27	Garofalo et al., 2009, 2011; Wei et al., 2017
miR-17/92	13q23.1	Oncogene	Up-regulation	NR2C2, HIC1, NR1I2, PTH, p38α, BCL2L11, PPP2R5E	Borkowski et al., 2015; Guinot et al., 2016; Baumgartner et al., 2018

### Tumor Suppressor miRNAs in NSCLC

#### let-7 Family

The let-7 family, a first known human miRNA (Pasquinelli et al., 2000; Reinhart et al., 2000), contains 10 isoforms that functions as tumor suppressors by targeting a wide variety of mRNAs encoding oncogenes (Roush and Slack, 2008), including RAS (Johnson et al., 2005). Takamizawa et al. reported that significantly shorter survival after potentially curative resection was observed in cases with reduced let-7 expression (Takamizawa et al., 2004). let-7c, a member of the let-7 family, prevents migration and invasion of NSCLC cells by degrading ITGB3 and MAP4K3. Low expression of let-7 was associated with metastasis, venous invasion, advanced TNM stages, and poor survival of NSCLC patients (Zhao et al., 2014). Loss of let-7 function enhances the lung tumor formation in mouse models, whereas exogenous delivery of let-7 to established tumors in mouse models of NSCLC significantly reduces the tumor burden (Trang et al., 2010). Let-7g effectively induces cell cycle arrest and cell death in K-Ras^G12D^ expressing murine lung cancer cells by targeting KRAS oncogene (Kumar et al., 2008). These evidences indicate that let-7 family may serve as prognostic marker and therapeutic target in certain type of NSCLC.

#### miR-34

The miR-34 family consists of miR-34a, miR-34b, and miR-34c, and is directly regulated by p53, a tumor suppressor gene (He et al., 2007). miR-34a is a direct proapoptotic transcriptional target of p53 that mediates part of the biological functions of p53. Down-regulated miR-34a expression is frequently seen in NSCLC, which may thus contribute to tumorigenesis by attenuating p53-dependent apoptosis (Raver-Shapira et al., 2007). Cortez et al. found that p53 regulates PDL1 expression by transcriptionally up-regulating miR-34 in NSCLC. miR-34 mimics alone or in combination with radiotherapy reduced PDL1 expression in the tumor and antagonized T-cell exhaustion (Cortez et al., 2015). MRX34, a liposomal formulation of miR-34a, is a potential first-in-class miRNA mimic for cancer therapy (Daige et al., 2014; Beg et al., 2017). Phase 1 study of MRX34 in patients with advanced solid tumors, including lung cancer, was conducted and demonstrated manageable toxicity in most patients and some clinical activity. Dose-dependent inhibition of related target genes provides proof-of-concept for miRNA-based cancer therapy (Hong et al., 2020).

#### miR-486

The low level of miR-486 was observed in various types of human cancer including NSCLC, and considered as an ideal biomarker in cancer diagnosis (Jiang et al., 2018). miR-486 directly targets components related to insulin growth factor (IGF) signaling, including IGF1, IGF1R, and PI3KR1, and functions as a tumor suppressor in NSCLC (Peng et al., 2013). *Pim-1*, a proto-oncogene, is up-regulated in NSCLC and its expression was associated with advanced stage and lymph node metastasis. miR-486 directly targets Pim-1 and downregulated miR-486 in NSCLC leads to the overexpression of Pim-1 to promote tumor progression, suggesting that critical tumor suppressive functions of miR-486 in NSCLC (Pang et al., 2014).

#### miR-218

Down-regulated miR-218 expression in NSCLC is implicated in histological grades and lymph node metastasis by targeting the EMT regulator, Slug and ZEB2 (Shi et al., 2017). Overexpression of miR-218 in NSCLC cells inhibits cell proliferation, invasion and colony formation by targeting the IL-6 receptor and JAK3. Down-regulated miR-218 expression was associated with poor prognosis of patients with NSCLC (Zhang et al., 2013; Yang et al., 2017). In addition, miR-218 functions as a tumor suppressor by targeting the expression of high mobility group box-1 (HMGB1) in NSCLC, suggesting that miR-218 might be a potential therapeutic biomarker for metastatic NSCLC patients (Zhang et al., 2013).

#### miR-200

The miR-200 family (miR-200a, miR-200b, miR-200c, miR-429, and miR-141) is a well-known tumor suppressor that inhibits EMT by targeting ZEB1 and ZEB2 in a wide range of cancers (Korpal et al., 2008; Park et al., 2008). Chen et al. found that ZEB1, an EMT activator and transcriptional repressor of miR-200, relieves miR-200 mediated repression of PD-L1 on tumor cells, leading to CD8+ T cell immunosuppression and metastasis (Chen et al., 2014). miR-200 mediated inhibition of ZEB1 sensitizes KRAS mutant NSCLC cells to MEK inhibition and reduces *in vivo* tumor growth of NSCLC (Peng et al., 2019). In addition, miR-200 family inhibits lung cancer cell invasion and metastasis by targeting Foxf2, a transcription factor that elevated in mesenchymal-like lung cancer cells (Kundu et al., 2016). miR-200 has also been reported to inhibit tumor angiogenesis in NSCLC by directly targeting interleukin-8 and CXCL1 secreted from the endothelial and cancer cells (Pecot et al., 2013), suggesting multifunction of miR-200 family in NSCLC progression and metastasis.

### Onco-miRNAs in NSCLC

#### miR-196b

The function of miR-196b in NSCLC is controversial. Our recent study demonstrated that the expression of miR-196b is up-regulated NSCLC, and is negatively regulated by RNA binding protein QKI-5, a tumor suppressor in NSCLC. miR-196b promotes NSCLC progression and metastasis by targeting the tumor suppressors, TSPAN12, GATA6, and FAS (Liang et al., 2020) (Huang et al., 2020). However, Tellez et al. reported that miR-196b-5p was epigenetically silenced in the premalignant stage of lung cancer, suggesting that tumor-suppressive functions of miR-196b in NSCLC (Tellez et al., 2016). These dual functions of miR-196b might be due to differences in stage and treatment received and differences in the ethnic origin of the analyzed patients. Further study needs to validate these results.

#### miR-221/222

The miR-221/222 Cluster is one of the most commonly upregulated miRNA clusters in various cancers (Di Leva et al., 2014). miR-221/222 plays a key role in tyrosine kinase inhibitors (TKIs) resistance in NSCLC. EGFR and MET regulate miR-221/222 expression to control gefitinib-induced apoptosis and NSCLC tumorigenesis by targeting apoptotic peptidase activating factor 1 (APAF1) (Garofalo et al., 2011). In addition, miR-221/222 is involved in TNF-related apoptosis-inducing ligand (TRAIL)-resistance and tumorigenesis of NSCLC by targeting PTEN and TIMP3 tumor suppressors (Garofalo et al., 2009). Another study also showed that exosomic miR-222-3p promoted cell proliferation, gemcitabine resistance, migration, and invasion of NSCLC by targeting SOCS3 (Wei et al., 2017).

#### miR-17/92 Cluster

The miR-17/92 cluster encodes six miRNAs, including miR-17, miR-18a, miR-19a, miR-20a, miR-19b-1, and miR-92a-1, and resides in intron 3 of the C13orf 25 gene at human chromosome 13q31.3, a region frequently amplified in various solid tumors (Ota et al., 2004). Hayashita et al. reported for the first time that miR-17/92 cluster is overexpressed in lung cancer cells and promotes cell growth (Hayashita et al., 2005). Inhibition of miR-17/92 cluster exerts cytotoxicity in p53-negative NSCLC cells by suppression of CYP24A1 (Borkowski et al., 2015). miR-19 family enhances Wnt signaling by targeting p38α in NSCLC, and increases NSCLC malignant potential (Guinot et al., 2016). Downregulated miR-19b expression is closely associated with reduced phosphorylation of ERK, AKT and their effector proteins in EGFR mutant NSCLC cells (Baumgartner et al., 2018), suggesting that targeting miR-19b could potentially be alternative therapeutic methods in EGFR mutant NSCLC.

#### miR-21

miR-21 plays oncogenic functions by suppressing many important tumor suppressor genes (Krichevsky and Gabriely, 2009). Large-scale profiling of miRNA expression in multiple human cancer tissue samples revealed that miR-21 is the only miRNA up-regulated in all types of analyzed tumor samples (Volinia et al., 2006). A high level of miR-21 is associated with advanced clinical stage and metastasis in NSCLC, and stimulates cell growth and invasion by inhibiting tumor suppressor PTEN in NSCLC (Zhang et al., 2010; Xue et al., 2016). The expression of miR-21 is increased and is associated with poor prognosis in NSCLC. miR-21 promotes tumorigenesis through inhibition of negative regulators of the Ras/MEK/ERK signaling pathway (Hatley et al., 2010), suggesting that promising oncogenic functions of miR-21 in NSCLC.

#### miR-224

Our previous studies demonstrated that miR-224 is up-regulated and promotes tumor progression and metastasis in NSCLC. miRNA-224-mediated tumor suppressor candidate 3 (TUSC3) deficiency enhances the metastatic potential of NSCLC through the regulation of three unfolded protein response pathways and HRD1-dependent endoplasmic reticulum associated degradation (ERAD) (Jeon et al., 2018). In addition, miR-224 directly targets tumor suppressor, TNFAIP1 and SMAD4, to promote tumor growth both *in vitro* and *in vivo* in NSCLC. Considering that SMAD4 plays a central role in the TGF-β family signaling pathways, and has low frequency of mutation and/or deletion, miR-224 might be an ideal therapeutic target for patients with certain NSCLC (Cui et al., 2015b).

NSCLC metastasis is a complex, multistep process involving a number of molecular and genetic changes, and is a sign of poor prognosis (Zhu et al., 2020). As described avobe, some miRNAs are closely implicated in fundamental processes of NSCLC metastasis. Therefore, deeply understanding miRNA signaling network will help to identify therapeutic targets for NSCLC metastasis.

## Circulating miRNAs in NSCLC

Circulating miRNA was initially discovered in the cell-free blood plasma and serum, and was considered as a novel class of biomarkers for diagnosis and prognosis of various diseases including cancer (Chen et al., 2008; Lawrie et al., 2008). The plasma miR-590-5p was significantly down-regulated in NSCLC patients and serves as a potential prognostic marker in NSCLC (Khandelwal et al., 2020). Hypoxic lung cancer-cell-derived extracellular vesicle miR-103a induces oncogenic M2 macrophages polarization by targeting PTEN, it results in activation of AKT and STAT3 signaling as well as expression of several immunosuppressive and pro-angiogenic factors to facilitate cancer progression (Hsu et al., 2018). Circulating miR-320a secreted from neutrophils of high-risk heavy-smokers induces immunosuppressive macrophages M2 phenotype through downregulation of STAT4 to increase lung cancer risk (Fortunato et al., 2019). Although a growing number of studies have reported that circulating miRNAs may serve as diagnostic and prognostic biomarkers in NSCLC, concordance between the experimental results is extremely low due to the lack of standardized measurement methods. Accordingly, establishing a standard guideline is extremely important to screening circulating miRNA biomarkers in NSCLC.

## Causes of miRNA Dysregulation in NSCLC

**Figure 1:**
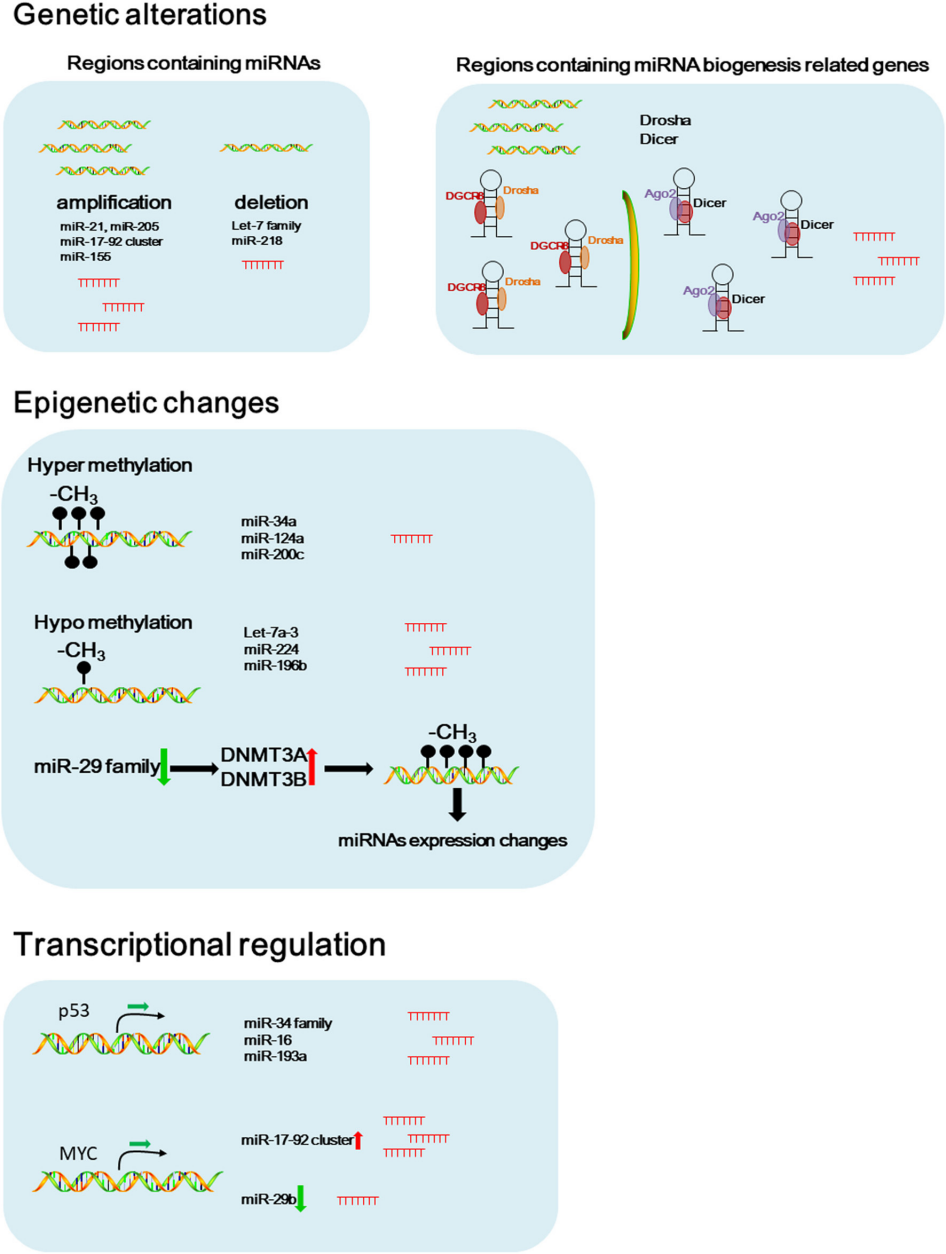
Potential causes of dysregulated miRNAs in NSCLC. Schematic figure of the possible pathways that involved in dysregulated miRNA expressions in NSCLC.

Aberrant miRNA signature was considered as an important biomarker for diagnosis and prognosis of NSCLC (Iorio and Croce, 2012a). Accumulating evidence suggests that genetic and epigenetic alterations, and transcriptional regulations are involved in miRNA dysregulation in cancers (Figure 1) (Iorio and Croce, 2012b).

### Genetic Alteration

Genetic alterations including chromosomal rearrangements, genomic amplifications, deletions, and gene mutations contribute to the miRNA dysregulation (Di Leva et al., 2014). In 2002, the earliest evidence of miRNAs deletion in human cancer was provided by Dr. Croce's group. They showed that the region containing miR-15a and miR-16-1 in chromosome 13q14 was frequently deleted in patients with B cell chronic lymphocytic leukemia (CLL) (Calin et al., 2002).

Amplification of miRNAs as a result of being located in amplified genomic regions is associated with carcinogenesis and tumor progression. miR-21 and miR-205, known as the oncogenic miRNAs, are located at the regions commonly amplified in lung cancer (Yanaihara et al., 2006). Another oncogenic miRNA, miR-17-92 cluster, is located within the third intron of the open reading frame 13 (C13orf25) at 13q31.3 genomic region. The region containing miR-17-92 cluster is frequently amplified in NSCLC, that results in upregulated miR-17-92 family expression contributing to NSCLC progression (Hayashita et al., 2005; Calin and Croce, 2006). A large scale copy number variation analysis using NSCLC tissue samples also showed that miR-21, miR-17, and miR-155 are the most frequently amplified miRNAs in NSCLC (Czubak et al., 2015). Inversesly, the let-7 family are located in regions commonly deleted in lung cancer, including let-7g at 3p21.1-21.2, let-7a-2 at 11q23-q24, and let-7c at 21q11.1 (Diederichs and Haber, 2006).

Aberrant expression or mutations in the genes that encoding key components of the miRNA biogenesis pathway contribute to the global downregulation of miRNAs in cancer (van Kouwenhove et al., 2011). Czubak et al. reported that the frequent amplification of DICER and DROSHA was observed in NSCLC (Czubak et al., 2015). In addition, the phosphorylation of argonaute 2 (AGO2) by c-Src inhibits the binding of DICER to AGO2 and promotes tumorigenesis of lung cancer, suggesting that change of these miRNA biogenesis related genes might cause alternation of miRNA signature in NSCLC (Liu et al., 2020). Under hypoxic stress, epidermal growth factor receptor (EGFR) suppresses the maturation of specific tumor-suppressor miRNAs by phosphorylation of argonaute 2 (AGO2). Phosphorylated AGO2 protein reduces the binding between Dicer and AGO2, results in inhibition of miRNA processing from precursor miRNAs to mature miRNAs in NSCLC cells (Shen et al., 2013).

### Epigenetic Changes

In addition to genetic alterations, epigenetic changes such as aberrant DNA methylation and histone modifications may contribute to the dysregulated miRNA expression in cancers (Egger et al., 2004).

Methylation in the miR-34a promoter region was frequently seen in various cancers including NSCLC, that results in down-regulated miR-34a expression (Lodygin et al., 2008). Hypermethylation in the promoter region of miR-124a significantly reduces miR-124a expression in lung cancer cells, results in increased expression of CDK6 and Rb phosphorylation (Lujambio et al., 2007). miR-200c plays a critical role in regulating EMT and inhibits cell invasion and metastasis. Hypermethylation in the promoter region of miR-200c significantly reduces miR-200a expression, and that results in induction of an aggressive, invasive, and chemoresistant phenotype in NSCLC (Ceppi et al., 2010).

Conversely, the promoter region of let-7a-3 gene was hypomethylated in lung adenocarcinomas, that results in elevated let-7a-3 expression and subsequent enhanced tumor phenotypic changes in lung cancer (Brueckner et al., 2007). Recently, our study reported that the hypomethylation in the CpG islands of the miR-224 promoter region increased the expression of miR-224, and promoted tumor progression and metastasis of NSCLC by targeting SMAD4 and TNFα-induced protein 1 (TNFAIP1) (Cui et al., 2015b). In another study, we found that the expression of miR-196b-5p was partially controlled by hypomethylation in its promoter region and up-regulated miR-196b-5p promoted NSCLC cell migration, proliferation, and cell cycle through directly targeting the tumor suppressors, GATA6 and TSPAN12 (Liang et al., 2020).

The miR-29 family, including miR-29a, miR-29b, and miR-29c, is a key regulator of DNA methyltransferases, DNMT3A and DNMT3B. In NSCLC, reduced miR-29 expression causes hypermethylation in the promoter region of some tumor suppressor genes by regulating DNMT3A and DNMT3B, leading to elevated expression of tumor suppressor genes (Fabbri et al., 2007).

In addition to DNA methylation, histone modifications play an important role in chromatin remodeling, and cooperate with DNA methylation to regulate miRNA expression in cancers (Chuang and Jones, 2007). KDM5B, a histone H3 lysine 4 (H3K4) demethylase, promotes epithelial-mesenchymal transition (EMT) of lung cancer cells by repressing the expression of the miR-200 family (Enkhbaatar et al., 2013).

### Transcriptional Control of miRNAs

Myc, an oncogenic transcription factor, has been reported to positively or negatively regulate the expression of many protein-coding genes and miRNAs (Croce, 2009). The overexpression of miR-17-92 cluster in NSCLC is associated with gene amplification of the miRNA cluster itself and enhanced expression of the myc gene (O'Donnell et al., 2005; Osada and Takahashi, 2011). In NSCLC, c-Myc suppresses miR-29b to promote tumor aggressiveness and poor outcomes by targeting tumor suppressor, FHIT (Wu et al., 2015).

p53, a well-known tumor suppressor, binds to the specific DNA sequence, termed the p53-responsive element (RE) to regulate p53 target genes. p53 promotes the transcription of the miR-34 family by binding to p53 REs in its promoter region (Bommer et al., 2007). p53 mediated increased expression of miR-34a promotes p53 induced apoptosis in NSCLC cells (Hollstein et al., 1991; He et al., 2007; Raver-Shapira et al., 2007). The upregulation of p53 simultaneously activates miR-34a and miR-16, which in turn targets Bcl2 to induce apoptosis in NSCLC cells (Upadhyay et al., 2019). Wang et al. found that miR-193a was directly activated by p53 at the transcriptional level and miR-193a targets EGFR through directly binding to 3′-UTR of the EGFR mRNA in NSCLC (Wang, W. et al., 2019). These facts indicate that the aberrant expression of transcription factors consistently contributes to the dysregulated miRNAs expression in NSCLC.

## miRNAs Contribute to Drug Resistance of NSCLC

Platinum-based chemotherapy is a standard first-line treatment for NSCLC patients. However, most patients with NSCLC eventually develop resistance to chemotherapy (Rizvi et al., 2016). The changes in the type and amount of miRNAs in exosomes are associated with the resistance of NSCLC cells to chemotherapeutic drugs (Zhao et al., 2015). Qin et al. demonstrated that the miRNAs were differentially expressed in the exosomes of cisplatin (CDDP)-resistant and CDDP-sensitive NSCLC cells. In CDDP-resistant NSCLC cell lines, the amount of miR-100-5p was significantly decreased in exosomes and was functionally involved in CDDP resistance of NSCLC (Qin et al., 2017). In addition, cancer-associated fibroblasts (CAFs) derived exosomes confer cisplatin resistance of NSCLC cells through transferring miRNA-130a (Zhang et al., 2021).

Aberrant EGFR signaling is the main cause of metastatic NSCLC. Molecular targeted therapy for EGFR in NSCLC has achieved certain effects. However, mutations in EGFR and feedback activation of other signaling pathways limited the therapeutic efficacy of targeted therapy (Lai-Kwon et al., 2021). Abnormal expression of miRNAs has been considered as one of the causes of tyrosine kinase inhibitors (TKIs) resistance (Calin and Croce, 2006). Inhibition of miR-483-3p promotes gefitinib resistance in EGFR-mutant NSCLC, suggesting that miR-483-3p is a promising target to overcome acquired EGFR TKI resistance in EGFR-mutant NSCLC (Yue et al., 2018). Garofalo et al. reported that miR-30b-c and 221-222 are involved in gefitinib, an EGFR inhibitor, resistance of NSCLC cells by modulating APAF-1 and BIM (Garofalo et al., 2011). Zhou et al. found that miR-34a restored the gefitinib sensitivity in HGF/MET activation-mediated gefitinib-resistant NSCLC cells by targeting MET (Zhou et al., 2014). These facts suggest that manipulating the expression of miRNAs could sensitize drug resistance in NSCLC.

## Conclusions and Future Directions

Recent advancements in whole-genome profiles of miRNAs for blood samples and biopsies of NSCLC patients facilitate discovery of new biomarkers for diagnosis, prognosis, aggressiveness, metastasis, and drug resistance of NSCLC. However, inconsistent results were also reported between different groups. These might be due to the differences in stage and treatment received for the analyzed samples. Thus, uniformed sample collection and detection methods, and adequate number of samples are necessary to obtain best results.

Preclinical studies of miRNA replacement therapy using miRNA mimics or inhibition of miRNA by antimiRs have shown promising results with minimal toxicity, indicating that targeting miRNAs might be potential novel therapeutics for NSCLC. Nevertheless, only few of miRNA therapeutics advanced into clinical trials. To achieve success in miRNA-based therapeutics, big challenge is identification of best target miRNAs in specific disease type. Other challenges include minimizing the off-target effects of miRNAs, preventing potential toxicities and developing more efficient and specific miRNA delivery methods.

## Author Contributions

RC conceived the structure of manuscript. XZ, MK, and XH collected the articles and made a table. HS and HT guide XZ and XH to collect articles and made a figure. MK and CC pre-revised the manuscript. RC and CC wrote the manuscript. All authors contributed to the article and approved the submitted version.

## Conflict of Interest

The authors declare that the research was conducted in the absence of any commercial or financial relationships that could be construed as a potential conflict of interest.
